# Measurement Properties of the Suicidal Behaviour Questionnaire-Revised in Autistic Adults

**DOI:** 10.1007/s10803-020-04431-5

**Published:** 2020-03-03

**Authors:** Sarah A. Cassidy, Louise Bradley, Heather Cogger-Ward, Rebecca Shaw, Erica Bowen, Magdalena Glod, Simon Baron-Cohen, Jacqui Rodgers

**Affiliations:** 1 School of Psychology, University of Nottingham, University Park, Nottingham, NG7 2RD UK; 2 The International Centre: Researching Child Sexual Exploitation, Violence and Trafficking, University of Bedfordshire, Luton, UK; 3 Autism Research Centre, University of Cambridge, Cambridge, UK; 4 Institute of Neuroscience, Newcastle University, Newcastle upon Tyne, UK; 5 Coventry and Warwickshire Partnership Trust, Coventry, UK; 6 College of Business, Psychology and Sport, University of Worcester, Worcester, UK; 7 Cambridge Lifetime Asperger Syndrome Service (CLASS), Cambridgeshire and Peterborough NHS Foundation Trust, Cambridge, UK

**Keywords:** Autism spectrum condition, Asperger syndrome, Autistic, Suicide, Suicidality, Self-harm, Measurement properties, COSMIN, Measurement invariance, Cognitive interview

## Abstract

We explored the appropriateness and measurement properties of a suicidality assessment tool (SBQ-R) developed for the general population, in autistic adults—a high risk group for suicide. 188 autistic adults and 183 general population adults completed the tool online, and a sub-sample (n = 15) were interviewed while completing the tool. Multi-group factorial invariance analysis of the online survey data found evidence for metric non-invariance of the SBQ-R, particularly for items three (communication of suicidal intent) and four (likelihood of suicide attempt in the future). Cognitive interviews revealed that autistic adults did not interpret these items as intended by the tool designers. Results suggest autistic adults interpret key questions regarding suicide risk differently to the general population. Future research must adapt tools to better capture suicidality in autistic adults.

## Introduction

Adults diagnosed with Autism Spectrum Conditions (ASC; hereafter autistic adults) are significantly more likely to report suicidal thoughts and suicidal behaviours (Cassidy et al. 2014, 2018c; Hedley and Uljarević 2018; Zahid and Upthegrove 2017) and to die by suicide (Hirvikoski et al. 2016; Kirby et al. 2019) compared to the general population. However, suicidality in autism is poorly understood and under researched (Cassidy and Rodgers 2017). In particular, there are few studies exploring *why* autistic people are more likely to contemplate and attempt suicide than the general population, to inform suicide prevention strategies for this group (Cassidy in press; Cassidy and Rodgers 2017; Hedley and Uljarević 2018). Addressing this crucial knowledge gap will require further research, but a key barrier is lack of validated research tools available to accurately capture suicidal thoughts and suicidal behaviours in autistic adults (Cassidy et al. 2018a; Hedley and Uljarević 2018). Therefore, it is unknown whether tools developed for, and validated in the general population operate similarly for autistic adults, or whether these tools need to be adapted for this group. This study therefore aimed to explore the appropriateness^1^ and measurement properties of a widely used and validated suicidality assessment tool originally developed for the general population, in autistic adults. This will in turn inform potential adaptations to better capture suicidal thoughts and suicidal behaviours in autistic adults in future research.

A previous systematic review showed that despite a growing number of studies exploring suicidality in autistic adults, none had yet used a tool with evidence of validity in this population, and no suicidality assessment tool had yet been developed or validated for autistic adults (Cassidy et al. 2018a). However, the review identified moderate-strong evidence in support of internal consistency, structural validity, and criterion validity for the Suicidal Behaviours Questionnaire-Revised (SBQ-R; Osman et al. 2001) in suicidality research in the general population (Cassidy et al. 2018a). The review found that the SBQ-R had been used in a number of research studies exploring suicidal thoughts and suicidal behaviours in the general population, but had not yet been extensively used or validated in clinical settings, or in psychiatric samples (Cassidy et al. 2018a). Importantly, the SBQ-R while brief and free to use had comparable quality of evidence for internal consistency, structural validity and criterion validity compared to other longer self-report and interview tools [i.e. the Beck Scale for Suicidal Ideation (BSS; Beck et al. 1988), and the Columbia Suicide Severity Rating Scale (C-SSRS; Posner et al. 2011)] that carry a high financial cost and are thus expensive to use in research (Cassidy et al. 2018a). The SBQ-R was therefore recommended as a validated suicidality assessment tool for use in general population research (Cassidy et al. 2018a). Hence, the SBQ-R could be a promising candidate tool to begin exploring the appropriateness and measurement properties of a suicidality assessment tool developed for and frequently used in research in the general population, in autistic adults (Cassidy et al. 2018a).

The SBQ-R is a four-item self-report questionnaire, assessing presence of lifetime suicidal thoughts and suicidal behaviours (item one), frequency of suicidal thoughts over the past year (item two), communication of threat of suicide attempt to others (item three), and likelihood of attempting suicide someday in the future (item four) (Osman et al. 2001). The characteristics of ASC could affect the measurement properties of this tool in autistic people in comparison to general population adults. For example, autistic adults often have difficulties in remembering what happened to them in the past (autobiographical memory) and imagining what will happen to them in the future (Crane et al. 2013; Lind and Bowler 2010). This could lead to difficulties particularly with item four (likelihood of future suicide attempt), and possibly items one and two (lifetime suicidality and frequency of suicidal thoughts over the past year). Autistic adults could also have difficulties in communicating their suicidality to others (item three) due to difficulties in identifying and describing their thoughts and feelings (termed Alexythymia; Bird et al. 2010), and differences in communication style (APA 2013) which is difficult for neurotypical people to interpret (Alkhaldi et al. 2019; Jaswal and Akhtar 2019; Mitchell et al. 2019; Sheppard et al. 2016). In addition to difficulties in communication, autistic adults may also have reduced opportunities to tell others about their suicidality, due to increased chance of being socially isolated (Hedley et al. 2018), and increased barriers to accessing appropriate diagnosis, treatment and support from mental health services (Au-Yeung et al. 2018; Camm-Crosbie et al. 2018; Crane et al. 2019).

Autistic people may thus interpret and respond differently to the SBQ-R items originally developed for the general population, which could be particularly problematic for research. For example, autistic people may tend to report reduced communication of suicide threat to others for reasons attributable to difficulties characteristic of autism, rather than reduced suicidality per se. This measurement difference could undermine the predictive power of this item in capturing suicidality in autistic adults compared to the general population. Such measurement differences could therefore result in reduced size of correlations between suicidality and other variables of interest in autistic people, lower internal consistency, and different factor structure, compared to the general population. Hence, it may not be valid to use tools such as the SBQ-R developed for the general population, to compare rates of suicidality between autistic and general population adults, as any group differences found could (at least in part) be attributed to measurement differences, rather than true differences in the variable (suicidality) under study.

Previous research has rarely explored the measurement properties of tools between groups (Cassidy et al. 2018a, b). This is highly problematic, as it is crucial when comparing mean scores on a tool between groups that both groups attribute the same meaning to the items, and all items measure the same construct (Byrne 2004). To explore this, measurement invariance analysis can quantitatively compare the structural equivalence of a tool between different groups or at different time points (Byrne 2004). Cognitive interviews (Willis and Artino 2013) can subsequently be used to explore how people within a group tend to interpret items in a tool, to check that items are interpreted as they were originally intended to capture the latent construct being measured, identify any problems in interpretation and inform changes to improve the relevance and clarity of items to a particular group. Given that no previous research has yet explored how autistic adults interpret and respond to suicidality assessment tools developed for and used in the general population, we use these two methods in combination to answer the following research questions:

1. Does a suicidality assessment tool (SBQ-R) validated in the general population similarly capture the latent construct of suicidality in autistic adults?
2. Do autistic adults interpret and respond to the SBQ-R questions as intended, and if not, how can the tool best be adapted to better capture suicidality in this group?

## Method

### Ethical Approval

The current study received ethical approval from the relevant local Psychology Research Ethics Committee (ethics approval reference P47603 and P45362), and was approved by both the Coventry Autism Steering Group, who provided feedback on the online questionnaire and cognitive interview schedule, and the scientific advisory group at the Autism Research Centre, University of Cambridge, prior to recruiting participants registered in Cambridge Autism Research Database.

### Design

The current study employed a sequential, explanatory mixed-methods design. First, an online survey quantitatively compared the factor structure of the SBQ-R between autistic and non-autistic adults. Second, a sub-sample of autistic adults from the online survey took part in qualitative semi-structure interviews, to explore their interpretation of the questions in the SBQ-R. We describe the methods for each stage of the study below.

## Online Survey

### Participants

The autistic group comprised 188 adults (40.4% male) who self-reported a diagnosis of ASC from a trained clinician, and a majority (80.9%) confirmed the clinic where this diagnosis was obtained. The general population group comprised 183 adults (33.9% male) who did not report a diagnosis of ASC, suspect they might be autistic, or report any autistic family members (to exclude those with the broader autism phenotype, Piven et al. 1997). Participants were aged between 18 and 70 years old. There were no significant differences in age [*t*(369) = 1.08, *p* = 0.282) or sex ratio (*χ*^2^(1) = 1.7, *p* = 0.192, OR 0.755, 95% CI 0.495–1.152] between the autistic and general population groups. The autistic group scored significantly higher on a validated self-report measure of autistic traits, the Autism Spectrum Quotient (AQ) (36.14, *SD* 8.16) than the general population group (19.93, *SD* 7.95) (*t*(331) = 0.657, *p* < 0.001) (Table 1).

**Table 1:** Participant characteristics

Variables	General population adult group	Autistic adult group
	Mean (SD)/%	Mean (SD)/%
Sex	33.9% male	40.4% male
Age (years)	40.92 (11.13)	39.66 (11.36)
AQ score	19.93 (7.95)	36.14 (8.16)
Employed	79.2%	48.1%
Satisfaction with living arrangements	78.11 (23.22)	69.04 (26.6)
Depression	47%	77.5%
Anxiety	37.7%	70.6%
≥ 1 Developmental condition	1.6%	23.4%
Unmet support	1.59 (1.56)	3.34 (2.4)
Age autism diagnosed	–	34.36 (13.34)

N.B. Unmet support needs = the total number of areas of support ideally liked − total number of areas support actually received, with larger values indicating higher number of unmet support needs (Cassidy et al. 2018c). Depression and Anxiety indicate self-reported diagnoses from a professional. Developmental conditions include Dyspraxia, Attention Deficit Hyperactivity Disorder, Developmental Delay, Learning Difficulty, Dyscalculia, Learning Disability, Other (Cassidy et al. 2018c)

### Measures

#### Autism Spectrum Quotient (AQ)

The AQ is a 50-item questionnaire assessing the number of self-reported autistic traits (Baron-Cohen et al. 2001). The AQ has been shown to reliably distinguish those with and without a diagnosis of ASC (Baron-Cohen et al. 2001; Ruzich et al. 2015) with scores ≥ 26 indicating potential diagnosis of ASC (Woodbury-Smith et al. 2005).

#### Suicidality

Participants completed the Suicide Behaviours Questionnaire-Revised (SBQ-R) (Osman et al. 2001), a four-item self-report questionnaire that assesses lifetime suicidal behaviour (on a scale from “Never” to “I have attempted to kill myself, and really hoped to die”), suicide ideation over the past 12 months (on a scale from “Never” to “Very Often (5 or more times)”), threat of suicide attempt (on a scale from “No” to “Yes, more than once, and really wanted to do it”), and likelihood of suicidal behaviour in the future (on a scale from “Never” to “Very likely”). The SBQ-R has been validated for use in general population samples to reliably distinguish people who have, from people who have not attempted suicide (Aloba et al. 2017; Osman et al. 2001). The SBQ-R is widely used in research with moderate-strong evidence in support of internal consistency, structural validity, and criterion validity in research with general population samples (Cassidy et al. 2018a).

#### Procedure

Data in the current study were extracted from the Mental Health in Autism Survey dataset, a large online survey co-designed in partnership with autistic adults, to explore their experiences of mental health, self-injury and suicidality (see Cassidy et al. 2018c). Participants were recruited to this study from research volunteers databases located in the University of Cambridge. Autistic adults and their family members across the UK and internationally register in the Cambridge Autism Research Database (http://www.autismresearchcentre.net/). General population adults without an autism diagnosis or family members with a diagnosis register in Cambridge Psychology (http://www.cambridgepsychology.com/login). Volunteers register in these databases to receive information about a variety of psychology research projects, and not mental health specifically. Additionally, participants were recruited from online adverts.

Autistic and general population adult participants, aged 18 years and over, without intellectual disability, were invited to complete an online survey about understanding and preventing mental health difficulties, self-injury and suicidality (see Cassidy et al. 2018c). Participants could take part regardless of prior experience of mental health difficulties or suicidality. Autistic participants who self-reported a clinical diagnosis of autism were included in the analysis. Participants read the participant information and indicated informed consent to participate via an online form. Participants were fully briefed about the nature of the research, that they could skip questions that made them feel uncomfortable and were provided information about relevant support services before and after taking part in the study. Participants subsequently completed questions on demographics, diagnoses (mental health, developmental conditions and ASC), non-suicidal self-injury, ‘camouflaging’ their autistic characteristics to ‘fit in’ in social situations, AQ, SBQ-R, current treatment (for mental health, self-injury or suicidality), and support (areas in which support was actually received and ideally liked but not yet received). Data pertaining to demographics, AQ, and SBQ-R are presented here, further information on the survey and dataset are available from a previous publication (Cassidy et al. 2018c).

#### Analysis Approach

Data were analysed using SPSS 24 and SPSS AMOS 21. Participants’ data with any missing values on the SBQ-R were excluded to allow item level analysis. Screening revealed no systematic pattern to the missing data, which accounted for a minority (9.8%) of the total dataset.

##### Confirmatory Factor Analysis

Before exploring the structural equivalence of the tool between groups, each group must first demonstrate adequate fit to a hypothesised model. There is strong evidence for the SBQ-R fitting a one factor solution in a general population sample (Aloba et al. 2017) as rated by a validated research tool (COSMIN) (Cassidy et al. 2018a). Therefore, a confirmatory factor analysis (CFA), using structural equation modelling, in AMOS, was conducted separately in the autistic and general population groups, and both groups combined.

We used established guidance from previously published research in assessing model fit: the χ^2^*/df* ratio should be close to zero (Bryant and Yarnold 1995), and values close to 0.06 on the root mean square of approximation (RMSEA) represent good fit (Hu and Bentler 1999), whilst values between 0.08 and 0.1 represent poor fit (Browne and Cudeck 1993). The comparative fit index should ideally be higher than 0.95, but values over 0.9 are considered acceptable (Hu and Bentler 1993), and Tucker-Lewis Index (TLI) values over 0.9 represent good fit (Browne 2014). The Chi-Square statistic (χ^2^) may be used as an indicator of fit (Browne 2014), but it is recommended to use this in combination with other goodness of fit indices, given that this statistic is greatly influenced by sample size (Stevens 2005).

##### Measurement Invariance Analysis

Measurement invariance analysis compares the structural equivalence of a tool between groups, using a multi-group confirmatory factor analysis approach (Byrne 2004; Byrne and Campbell 1999). A series of nested models are tested, with increased constraints on the model, to assess evidence for increasingly strict levels of measurement invariance between groups (Byrne 2004; Cheung and Rensvold 2002).

First, the configural invariance model has no equality constraints, and assesses whether sets of items measure the same latent variable (i.e. suicidality) in both groups. Second, metric invariance constrains the factor loadings, and assesses whether the strength of the relationship between items are the same for both groups. Subsequently, the source of any metric non-invariance can be isolated to particular item(s) which are not similarly associated with the underlying construct being measured in each group. Evidence for metric non-invariance suggests that each group attributes a different meaning to particular item(s). Third, scalar invariance constrains the intercepts of items, and assesses whether total scores on the scale consist of similar individual item scores in each group. Fourth, residual invariance constrains model item unique variance between the groups, and assesses whether the scale items measure the latent constructs with the same amount of measurement error.

## Cognitive Interviews

Cognitive interviews ensure that the target population interpret and respond to questions as the researchers intend, a crucial part of establishing content validity for a tool (Willis et al. 2005). Lack of evidence for measurement invariance, would suggest that groups do not attribute the same meaning to the items. However, measurement invariance analysis does not specify *how* the items are interpreted differently by a group, to help interpret differences in item level scores between groups, or inform adaptations to the SBQ-R.

### Participants

A sub-group of 15 autistic adults (8 male; 7 female) who completed the online survey were invited to complete a cognitive interview while completing the SBQ-R. Participants were randomly selected from the wider online sample.

### Cognitive Interview Schedule Development

It is crucial to prepare a comprehensive set of pre-prepared prompts to identify and clarify problems in interpreting the questions in a tool (Willis et al. 2005). To develop this, three patient and public involvement (PPI) focus groups were convened as part of a public engagement event which discussed how to adapt mental health assessment tools for autistic adults (none of whom took part in the cognitive interviews). Each focus group consisted of seven participants: one facilitator, and equal representation of autistic adults, clinicians and researchers in each group. Each member was given a copy of the SBQ-R, and the facilitator asked the group to discuss any potential problems autistic adults may have in interpreting the questions, and how these could be addressed. Each facilitator compiled up to five key points which were subsequently presented to all attendees for further discussion in a plenary session, with any additional points noted by the plenary chair. A subsequent focus group was held with the Coventry Autism Steering Group, who further discussed the main points raised at the public engagement event and provided feedback on the researcher’s draft interview schedule. This ensured that the pre-prepared prompts were comprehensive, relevant and clear to autistic adults. Please see Table 2 for a summary of the discussion points from the PPI focus groups and associated pre-prepared prompts developed for the cognitive interview schedule.

**Table 2:** Summary of key discussion points from the PPI focus groups, and examples of pre-prepared prompts designed to explore these issues

SBQ-R Item	PPI Feedback	Examples of pre-prepared prompts
Overall	The tool is reliant on verbal processing as a self-report measure It is difficult to ascertain the difference between response options (likely, mostly, never etc.) There are too many response choices and it is difficult to select the appropriate response It is unclear what the scale is measuring. What ‘counts’ as a suicidal thought or behaviour?	What were you thinking about when you first saw the questionnaire? What do you think about the layout of the questionnaire? How relevant were the questions to you? Where there any important topics or areas missing? What was the most important question to you?
Have you ever thought about or attempted to kill yourself?	This question is unclear and asks two different things Is it unclear what “plan suicide” means here The timescale is unclear (is it current or lifetime?)	What do you think about the language of this question? Was the question easy or difficult to answer? What does ‘have you ever …’ mean to you? What does ‘Never/a brief passing thought’ mean to you? What does ‘really wanted to/did not want to/really hoped to’ mean to you?
How often have you thought about killing yourself in the past year?	How can “5 times” be considered “often” and the maximum response for this question? The scale is unclear	What do you think about the language of this question? Was the question easy or difficult to answer? What does ‘how often/past year’ mean to you? What does ‘Never/rarely/sometimes/often/very often’ mean to you?
How likely is it that you will attempt suicide someday?	It is difficult to ascertain differences in response options between mostly, never and no chance at all	What do you think about the language of this question? Was the question easy or difficult to answer? How relevant is this question to you? What does ‘how likely’ mean to you? What does ‘Never/No chance at all/Rather unlikely/Unlikely/Likely/Rather Likely/Very likely’ mean to you? What does ‘Someday’ mean to you? What time-period were you thinking of?
Have you ever told someone that you were going to commit suicide or that you might do it?	This question is unclear and asks two different things The question is not autism relevant—autistic people may not routinely communicate suicidal thoughts or plans to others, but still experience them “Commit” suggests a moral judgement	What do you think about the language of this question? Was the question easy or difficult to answer? How relevant is this question to you? What does ‘have you ever …’ mean to you? What does ‘really wanted to/did not want to/really hoped to’ mean to you?

#### Procedure

A combination of approaches were used to identify and explore problems autistic adults experienced when trying to interpret and respond to the SBQ-R (Willis et al. 2005, 1999). First, a “think aloud” approach was used while autistic adults completed the SBQ-R. To introduce participants to the think aloud procedure, they were asked: “Think about how many windows there are in your house. As you count up the windows, tell me what you are seeing and thinking about”. All participants were able to complete this task. Participants were subsequently asked “tell me what you are reading and thinking about when you fill in this questionnaire”. Two researchers silently took notes during the “think aloud” phase, and key points were subsequently followed up in the semi-structured interview using the pre-prepared prompts. All interviews were audio recorded, and later transcribed for analysis.

### Analysis Approach

Although cognitive interviews utilise qualitative analysis techniques to identify patterns from the interview data, there are key differences to traditional qualitative analysis approaches. First, the aim of the cognitive interview is much more specific, exploring the cognitive processes underlying responses to each item on a tool. Thus, the analysis is conducted item by item, rather than across the whole data corpus. Two researchers (SAC and LB) made notes during each interview identifying particular problems participants experienced when answering each question during the interview, which were discussed and combined in a debrief session immediately after each interview. These notes were then checked against the transcription by LB. Further, a researcher not involved in any of the interviews HCW read each transcript independently, and noted the main issues identified for each question. A consensus meeting was held between the researchers to ensure consistent issues were identified.

## Results

### Online Survey

#### Confirmatory Factor Analysis

In the autistic, general population, and combined groups, the single factor model (Aloba et al. 2017) was tested. Fit indices all fell within recommended parameters indicating good overall fit of the model in each group (Table 3).

**Table 3:** Model fit of Confirmatory Factor Analysis in separate and combined groups

Model	*N*	*Χ*^*2*^	*df*	*Χ*^*2*^/*df* ratio	*p*	RMSEA	CFI	TLI
General population adult	183	2.53	2	1.263	.283	.038	.998	.993
Autistic adult	188	.62	2	.311	.733	.001	1	1.02
General population and autistic adults	371	2.11	2	1.054	.349	.012	1	.999

Recommended goodness of fit indices values demonstrating good model fit: χ^2^/df ratio close to zero (Bryant and Yarnold 1995), RMSEA < 0.06, CFI > 0.95 and TLI > 0.9 (Browne 2014; Hu and Bentler 1993)

*RMSEA* root-mean-square error of approximation, *CFI* Comparative Fit Index, *TLI* Tucker–Lewis Index

#### Measurement Invariance Analysis

As specified above, the series of nested models were tested, assessing both absolute model fit and comparative fit. Significant degradation in fit indicates lack of evidence for measurement invariance between groups (Table 4). First, the configural invariance model showed good fit. Therefore analysis proceeded to the next step, constraining the factor loadings, to assess evidence for metric invariance between the groups. The metric invariance model showed good fit, but chi-square analysis showed a significant degradation in fit compared to the configural (baseline) model. Additionally, the degradation in RMSEA and CFI were above recommended levels of degradation in fit (delta CFI < 0.01, delta RMSEA < 0.015) (Chen 2007). This suggests lack of evidence for metric invariance between groups, and thus further levels of stricter invariance were not tested.

**Table 4:** Results of tests for invariance in SBQ-R across the autistic and general population adult groups

Model	*Χ*^*2*^	*df*	Model Fit	CFI	TLI	ΔM	Model difference		*p*
			RMSEA				Δdf	Δ*χ2*	
M1: Configural invariance (unconstrained)^a^	3.15	4	.001	1	1				.534
M2: Weak factorial/metric invariance	15.39	7	.057	.981	.967	M2–M1	3	12.25**	.007
Source of metric non-invariance									
M3: Item 2 constrained	5.49	5	.016	.999	.997	M3–M1	1	2.34	.126
M4: Item 3 constrained	6.88	5	.032	.996	.99	M4–M1	1	3.73*	.053
M5: Item 4 constrained	14.59	5	.072	.978	.948	M5–M1	1	11.44***	.001
Source of metric non-invariance (Byrne 2004 rigorous method)									
M6: Item 2 + 3 constrained	7.64	6	.027	.996	.993	M6-M1	2	4.49	.106
M7: Item 2 + 3 + 4 constrained	15.93	7	.057	.981	.967	M7-M1	3	12.25**	.007

*RMSEA* root-mean-square error of approximation, *CFI* Comparative Fit Index, *TLI *Tucker–Lewis Index

*p = .05, **p < .01,***p < .001

^a^Significant degradation in fit is seen after this model

To isolate particular metric non-invariant item(s) between groups, factor loadings were constrained at the item level (Table 4) (Byrne 2004). Two sets of models were run. The first set constrained one item separately in each step, which is considered a more lenient test of item level metric invariance (Byrne 2004). The second set added in item constraints for previous item(s) which were shown to be measurement invariant, as a stricter test of item level metric invariance (Byrne 2004).

The first set of models showed that constraining the factor loading for item two did not result in a significant degradation in fit, suggesting measurement invariance. There was a significant degradation in fit when separately constraining items three and four, suggesting measurement non-invariance for these items using a more lenient test.

To ascertain whether items three and four were likely both metric non-invariant between groups, two stricter models were run, including previous items which were found to be measurement invariant. Constraining both items two and three did not result in a significant degradation in fit, suggesting that item three is in fact measurement invariant under a stricter test of item level measurement invariance. Constraining items two, three and four resulted in a significant degradation in fit, suggesting that item four remained measurement non-invariant in the stricter test of item level measurement invariance (Table 4).

#### Item Comparisons

Given lack of evidence for metric invariance between the groups, total scores on the SBQ-R could not be compared between the groups. Therefore, scores on individual items were compared between autistic and general population adults. Results showed that autistic adults scored significantly higher than general population adults on each item of the SBQ-R with large effect (Table 5).

**Table 5:** Item level factor loadings and item comparisons between the autistic and general population adult groups

SBQ-R Item	Autistic group		General population group		Item Comparisons
	Mean (SD)	Factor loading	Mean (SD)	Factor loading	
1. Lifetime suicidality	3.03 (.93)	.785	2.2 (.95)	.835	*t*(369) = 8.55, *p* < .001, *d* = .88
2. Frequency of suicidal ideation in past year	2.95 (1.64)	.805	2.04 (1.44)	.808	*t*(369) = 5.72, *p* < .001, *d* = .59
3. Threat of suicide attempt	1.75 (.86)	.689*	1.28 (.61)	.712	*t*(369) = 6.05, *p* < .001, *d* = .63
4. Self-reported likelihood of suicidal behaviour in the future	2.54 (1.68)	.862**	1.43 (1.32)	.803	*t*(369) = 7.02, *p* < .001, *d* = .73

*p = .05

**p < .01.

### Cognitive Interviews

#### Item One: “Have You Ever Thought About or Attempted to Kill Yourself?”

Many participants (9) described difficulties with item one, with the most common being that the options did not reflect their experience (6 participants). Individual participants reported “something missing” [PT4] between response options two and three a/b “a brief passing thought and I’ve had a plan” [PT6], because it might be that you have “a bit more than a passing thought but you haven’t actually got a firm plan” [PT1]. This detail was considered important (3 participants), because “you can attempt to kill yourself without formulating a plan” [PT1]. Participants also reported, “a lot of words are open to interpretation” (PT4), for example, the word ‘plan’ caused difficulty because it required participants to interpret “what is a plan?” [PT7]. Whilst another questioned the longevity of a plan in regard to the wording ‘had a plan’ because, “if you have a plan … isn’t that good for all … eternity” [PT1]. Options were described as being “really very similar” [PT4] and one participant reported that “more than one applies” [PT2], particularly in relation to sub-items which distinguish intent (i.e. 3a/3b and 4a/b which delineate “did not want to die” and “really wanted”/”really hoped to” die).

#### Item Two: “How Often Have You Thought About Killing Yourself in the Past Year?”

Many participants (9) also described difficulties in answering item two, as the response options once again did not reflect their experience. The most common difficulty was with the scale because the “numbers are pretty low” [PT6]. One participant reported that for response option five “Very Often (5 or more times)”, “five or more times, doesn’t seem a lot” [PT10] which made them question their own ideation, “does that mean I am really bad?” [PT10]. Intensity or seriousness of the suicidal thought was also not captured (6 participants) as one participant needed more information in order to answer the question, “Is it just a brief passing thought or [is] it … more serious?” [PT1].

#### Item Three: “Have You Ever Told Someone that You were Going to Commit Suicide or that You Might Do It?”

Many participants (6) questioned the relevance of this question, with individual participants saying it was “irrelevant” [PT1], “abstract” [PT7] or were not sure “why it matters” [PT10], as they felt they had “no one to tell” [PT1] or had “never told someone” [PT4]. Participants also reported that the question was “illogical” [PT10], as it contained “two questions” [2 participants] both of which “mean two different things” [PT7]. Participants also questioned the sensitivity of this question (7), as “commit suicide is not a good phrase” [PT4] because “suicide isn’t a crime, so you don’t commit it, you take your own life” [PT2].

#### Item 4: “How Likely it is that You Will Attempt Suicide Someday?”

The majority (11 participants) described difficulties in interpreting and answering this question. Some reported difficulties with the scale (6 participants), with individual participants reporting it to be “nonsense” [PT1] as many of the different response options were very similar: “never or no chance at all is the same thing … as is rather unlikely and unlikely” [PT6]. Participants also had difficulty with the word ‘someday’ (5 participants), which could mean “next week or thirty years from now” [PT12], and thus required them to “predict the future” [PT11], which made the question “almost unanswerable” [PT8], as “it depends” [PT9].

## Discussion

The current study aimed to explore the appropriateness and measurement properties of a widely used suicidality assessment tool validated for use in general population research, the SBQ-R (Osman et al. 2001) in autistic adults. Despite a growing body of research showing increased risk of suicidal thoughts and suicidal behaviours in autistic adults (Cassidy et al. 2014, 2018c), there is no suicidality assessment tool yet validated for this group (Cassidy et al. 2018a; Hedley and Uljarević 2018). The cognitive style and experiences of autistic people may affect the interpretation and thus validity of suicidality assessment tools such as the SBQ-R (Cassidy et al. 2018a). For example, communication differences (Alkhaldi et al. 2019; Jaswal and Akhtar 2019; Mitchell et al. 2019; Sheppard et al. 2016), lack of social connections (Cassidy in press; Cassidy et al. 2019; Hedley et al. 2017; Orsmond et al. 2013; Pelton and Cassidy 2017; Pelton et al. in press) and alexithymia (Bird et al.. 2010) could all result in reduced endorsement of communicating suicide threat to others, without necessarily indicating decreased experience of suicidality (Cassidy et al. 2018a). Difficulties in abstract future thinking in autistic people (Crane et al. 2013; Lind and Bowler 2010) could result in difficulties when rating one’s likelihood of attempting suicide ‘someday’ in the future (Cassidy et al. 2018a).

Consistent with our hypotheses, results suggest that the SBQ-R does not operate in the same way in autistic and general population adults. First, there was evidence for metric non-invariance for item four (likelihood of a future suicide attempt), with a significantly higher factor loading compared to the general population. This suggests that asking about likelihood of a future suicide attempt ‘someday’ appears to be more strongly associated with the underlying construct (suicidality) in the autistic compared to the general population group. There was also evidence of measurement non-invariance for item three (communication of threat of suicide attempt), with a lower factor loading in the autistic compared to the general population group. This suggests that asking about whether a person has told anyone else about their suicidality is less strongly associated with the underlying construct (suicidality) in the autistic compared to the general population group. Results thus suggest that autistic and general population adults attribute different meaning to these items of the SBQ-R, meaning that scores cannot be compared between these groups, or interpreted in line with the current clinical cut-off identified in the general population.

Cognitive interviews explored how autistic adults interpreted and responded to each item of the SBQ-R, to help interpret results from the above measurement invariance analysis. Many autistic adults reported the SBQ-R questions were difficult to interpret and respond to, were not autism relevant, and did not capture their experience of suicidality as autistic people. Item one did not capture serious, intense suicidal thoughts that occur in absence of a plan that could lead to spontaneous suicide attempts when lethal means of self-harm are available in the moment. Item two did not sufficiently capture the full range of frequency, duration and intensity of suicidal thoughts in the past year. Item three was considered irrelevant by many autistic people, given lack of social connections and opportunities to tell others about their suicidal intent. Item four was considered the hardest question to answer given the ambiguity of the term ‘someday’ and difficulty for many autistic adults for ‘predicting the future’.

Results from the cognitive interviews are consistent with the findings from the measurement invariance analysis, and provide important context for interpreting item level measurement differences between autistic and general population adults. For example, autistic people may lack social connections and opportunities to disclose suicide intent to others, but still experience suicidal intent. Therefore, this item may not be as strongly associated with other suicidality items (lifetime, current and future suicidal thoughts and behaviours), as in the general population. This is reflected in the lower factor loading for item three (communication of suicidal intent) in the autistic compared to the general population. Whereas for item four (likelihood of attempting suicide someday in the future) despite the difficulty in answering an ‘impossible’ future question, and having to choose a response in a ‘grey area’, this item is nevertheless more strongly correlated with the other suicidality items in autistic people compared to the general population. This is reflected in the significantly higher factor loading for item four in the autistic compared to the general population group.

These findings provide important and novel insights into the potentially unique nature of suicidality in autism, and how to adapt current tools to more accurately identify suicidality in this group. For example, many participants described their experiences of attempting suicide in the absence of a plan when lethal means presented themselves in a moment of crisis, and difficulty in understanding the concept of a suicide plan. Future research must further explore whether the phenomenology of suicide attempts in autism is different to the general population. For example, whether suicide attempts without a plan are more common in autistic people, or more driven by availability of access to lethal means of self-harm.

In adapting the SBQ-R, it will be important to not only consider results from our research, but results from studies that have adapted survey tools for autistic adults. Similar to the findings in our study, autistic adults tend to report difficulties with complex language, imprecise response options, lack of autism relevant items, and inappropriate or insensitive language (e.g. Nicolaidis et al. 2020). Therefore, it will be important to avoid multi-clause questions, include questions about the intensity and frequency of suicidal thoughts without a plan evident, and provide a more concrete alternative to gauge future suicide intent. Communication of suicidal thoughts and behaviours to others although difficult for autistic people due to lack of social connection and difficulty in social and communication skills, nevertheless appears to be a potentially important indicator and correlate of the underlying construct of suicidality in this group (although not as strong compared to the general population). Rather than excluding such items, it will be important clinically to explore not only whom the autistic person has disclosed their suicidality to (e.g. online, to a friend, or healthcare provider etc.), but also why the person may not have wanted or been able to disclose their suicidality to others (e.g. lack of support or contact with services, social isolation, difficulty in communicating one’s feelings, fear etc.). Probing the context and reasons for communicating suicidal thoughts and behaviours to others will be important to inform treatment, support and suicide safety planning for autistic people experiencing suicidal thoughts and behaviours.

The current study has a number of strengths and limitations. A key strength was the participatory approach to the study. Feedback from autistic people and those who support them ensured that the online survey, and cognitive interview schedule, were appropriate and accessible to autistic adults who took part, and comprehensively explored how autistic adults interpreted and responded to the items of the SBQ-R. A further strength was the mixed methods approach, which allowed us to explore the structural equivalence of a tool validated for the general population in comparison to autistic adults, and explore in more depth the root causes of any measurement non-invariance. Limitations of the current study are that the results are only relevant to autistic adults without intellectual disability (ID), who were diagnosed in adulthood—a particularly high-risk group for suicide (Cassidy et al. 2014, 2018c; Hirvikoski et al. 2016). It will be important to explore how tools can be adapted for autistic children, and autistic people with ID, where self-injurious behaviour are common (Minshawi et al. 2014) and it is unclear whether this is indicative of suicidality. Only 40% of the autistic group in the current study was male, which is lower than in the wider autistic population (Dworzynski et al. 2012). Autistic females without co-occurring intellectual disability are also more at risk of dying by suicide than non-autistic females (Hirvikoski et al. 2016; Kirby et al. 2019). This could limit the generalisability of our results to the wider autistic population. However, in the current study both groups had a similar gender ratio, meaning that measurement differences between the autistic and general population groups are not attributable to differences in gender. Given possible differences in suicide risk between autistic men and women, future research exploring the measurement properties of adapted autism specific suicidality assessment tools should test for measurement invariance between autistic men and women. However, this was beyond the scope of the current study. The sample size used in the cognitive interviews was in line with recommendations (Willis et al. 2005) and the researchers agreed that saturation point (where no new information emerged from further interviews) was reached.

Research has suggested that suicidality assessment tools on the whole are poor predictors of future suicide attempts, many perform worse than patient self-report or clinician opinion, and may therefore be a waste of valuable resources (Quinlivan et al. 2016, 2017). In light of such evidence, our focus on exploring the appropriateness and measurement properties of such an assessment tool in autistic people could be questioned. However, validity of tools vary according to context in which they are applied and the purpose they are used for (Kamphaus and Frick 2005). Our previous systematic review showed that the SBQ-R had strong evidence in support of its measurement properties specifically for use in research, for example to distinguish sub-groups of people who have attempted suicide from people who have not attempted suicide (Cassidy et al. 2018a). Hence, our results are most relevant for research, and future research will need to ascertain the best methods of assessing risk of future suicide attempts in autistic people. Further, given the lack of any validated tools or data regarding how autistic people may interpret questions attempting to probe suicidality, in light of the significantly increased risk of death by suicide in this group (Hirvikoski et al. 2016; Kirby et al. 2019), it is imperative to obtain these data to inform more accurate and useful questions for use in research and clinical practice. It is also important to highlight that such tools should form a start point, as part of a full psycho-social assessment when assessing suicide risk, and not be relied upon in isolation to inform any clinical judgment, particularly regarding access to treatment or support in any group.

Results from the current study suggest that a suicidality assessment tool widely used in the general population, the SBQ-R, cannot be directly compared with autistic people in research or used in clinical practice without adaptation for this group. In light of our results from the current study, our group is adapting the SBQ-R in partnership with autistic adults, with the aim of better capturing suicidality in this group. Our research joins an important call to action to explore suicidality in autism (Cassidy and Rodgers 2017), and develop new validated tools which more accurately capture the unique presentation of mental health problems and suicidality in this group for use in future research and clinical practice (Cassidy et al. 2018a, b; Wigham and McConachie 2014).

## Footnotes

1 “Appropriateness” in the context of the current study refers to the clarity and relevance of items in a tool to the group(s) in which the tool is intended to be used.
